# Cognitive aging and reserve factors in the Metropolit 1953 Danish male cohort

**DOI:** 10.1007/s11357-024-01427-2

**Published:** 2024-11-21

**Authors:** Mostafa Mehdipour Ghazi, Olalla Urdanibia-Centelles, Aftab Bakhtiari, Birgitte Fagerlund, Mark Bitsch Vestergaard, Henrik Bo Wiberg Larsson, Erik Lykke Mortensen, Merete Osler, Mads Nielsen, Krisztina Benedek, Martin Lauritzen

**Affiliations:** 1 Pioneer Centre for AI, Department of Computer Science, Faculty of Science, University of Copenhagen, Copenhagen, Denmark; 2 Department of Clinical Neurophysiology, Copenhagen University Hospital, Copenhagen, Denmark; 3 Department of Clinical Physiology and Nuclear Medicine, Glostrup University Hospital, Glostrup, Denmark; 4 Department of Psychology, Faculty of Social Sciences, University of Copenhagen, Copenhagen, Denmark; 5 Child and Adolescent Mental Health Center, Mental Health Service, Capital Region of Denmark, Copenhagen, Denmark; 6 Department of Clinical Medicine, Faculty of Health and Medical Sciences, University of Copenhagen, Copenhagen, Denmark; 7 Department of Public Health, Faculty of Health and Medical Sciences, University of Copenhagen, Copenhagen, Denmark; 8 Center for Clinical Research and Prevention, Bispebjerg & Frederiksberg Hospital, Copenhagen, Denmark; 9 Department of Neurology, Zealand University Hospital, Roskilde, Denmark; 10 Department of Neuroscience, Faculty of Health and Medical Sciences, University of Copenhagen, Copenhagen, Denmark

**Keywords:** Cognitive decline, Cognitive reserve, Machine learning, Aging risk factors, Magnetic resonance imaging, Electroencephalography

## Abstract

Identifying early predictors of cognitive decline and at-risk individuals is essential for timely intervention and prevention of dementia. This study aimed to detect neurobiological changes and factors related to cognitive performance in the Metropolit 1953 Danish male birth cohort. We analyzed data from 582 participants, aged 57–68 years, using machine learning techniques to group cognitive trajectories into four clusters differentiating high- and low-performing groups. These clusters were then evaluated with MRI, EEG, and lifestyle/familial risk factors to identify predictors of cognitive decline. Low education and occupation, alcohol consumption, and type 2 diabetes were associated with lower cognitive performance. Declines in neocortical volume and increases in frontotemporal alpha and temporoparietal gamma activity preceded clinical symptoms of cognitive decline. Neocortical atrophy and disruptions in network activity were prominent in lower-performing groups, with higher education and IQ scores and a lower prevalence of lifestyle factors moderating cognitive decline.

## Introduction

Aging is associated with significant variability in cognitive and neurobiological changes both within individuals and across populations. Longitudinal assessments of cognitive aging seek to clarify the neural network capacity, efficiency, and flexibility that underlie such changes. These studies aim to delineate the timeline and patterns of cognitive decline across various domains, particularly to distinguish pathological aging from normal aging. Notably, deficits in associative learning and memory have been consistently identified as early indicators of pathological cognitive decline. Associative learning, as the ability to form connections between unrelated stimuli or ideas, is essential for complex cognitive processes including memory formation. Hence, the processing speed of cognitive information differs among individuals and typically declines with age. However, education, occupational complexity, and mental activities can help the brain remain resilient against aging or pathology. This cognitive reserve helps maintain cognitive function over time, likely through more efficient neural processing or the recruitment of alternative neural networks.

Recent studies have emphasized the predictive value of processing speed and working memory in the early detection of Alzheimer’s disease and related cognitive decline [1–5]. The paired-associate learning (PAL) task from the Cambridge Neuropsychological Test Automated Battery (CANTAB) has proven especially effective, demonstrating high sensitivity and specificity in predicting conversion from questionable dementia to dementia of the Alzheimer’s type (DAT) [6–8]. It remains predictive even when other neuropsychological tests and dementia screening tools like the Mini-Mental State Examination (MMSE) fail to detect impairment [9–11]. Research suggests that PAL not only helps distinguish early-stage Alzheimer’s from other dementias [12] but also from conditions like depression [13].

Verbal associative learning and memory measures have been useful in differentiating DAT and prodromal DAT from healthy controls, supporting their utility in clinical diagnosis [14–17]. Cognitive domains beyond learning and memory can also be impacted in prodromal DAT patients [18], and including measures of processing speed in high-risk individuals has been shown to significantly enhance predictive power for the later onset of DAT compared to memory tasks alone [19]. More specifically, increased reaction times can differentiate between DAT, questionable dementia, depression, and healthy controls and are related to subsequent declines in MMSE scores [13]. Besides, working memory plays a role in predicting the progression from questionable dementia to DAT [10], correlating with later declines in MMSE performance [13]. While sustained attention declines slightly with normal aging, it deteriorates more significantly in DAT cases [20]. However, research remains divided on the timeline of cognitive decline in aging with and without disease [21, 22].

The Nun study [23] suggests that increased linguistic complexity in youth serves as a protective factor against age-related cognitive decline and clinical symptoms, even when neuropathology is present. This finding underscores intelligence’s modulating effect on normal aging and dementia. The brain can recruit alternative neural networks to compensate and maintain function despite neurodegeneration. This cognitive reserve is driven by factors such as genetically derived innate cognitive abilities [24], education [25, 26], and lifelong cognitive activities [27, 28]. Robust evidence suggests that cognitive reserve can delay the clinical symptoms of neurodegeneration, though the subsequent functional decline may be steeper once symptoms manifest [29, 30]. Additionally, cognitive and physical activities may have neuroprotective effects, potentially enhancing structural plasticity [28, 31, 32], possibly by upregulating brain-derived neurotrophic factor [33].

**Fig. 1:**
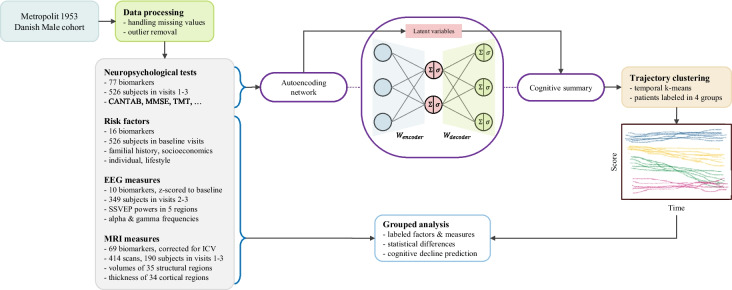
Overview of the methodological workflow used to study the Metropolit cohort, illustrating the steps in detecting cognitive aging and identifying reserve factors. The figure summarizes the key stages of data processing, clustering, and analysis, including the integration of cognitive, MRI, and EEG data, as well as the assessment of associated risk factors

In this study, the cognitive reserve influenced by education and lifestyle factors such as alcohol use and type 2 diabetes is hypothesized to delay age-related cognitive decline in the Danish Metropolit 1953 male cohort. This cohort was derived from 11,591 boys born in the Copenhagen Metropolitan area including a comprehensive set of data on cognitive performance, social background, and health collected since 1965 [34]. Between 2010 and 2022, cohort members underwent new rounds of cognitive testing, electroencephalography (EEG), magnetic resonance imaging (MRI), and blood tests to identify early subclinical predictors of cognitive decline. We employ machine learning models to examine multivariate longitudinal data from the Danish Metropolit cohort, testing the hypothesis that cognitive reserve can mitigate the clinical impacts of cognitive decline. Recent advances in machine learning have highlighted brain activity, physical exercise, and motivation as key factors that preserve cognitive function in aging [35]. These techniques offer reliable tools for identifying subtle patterns and interrelations between different phenotypes in complex healthcare datasets and selecting biomarkers that best predict cognitive outcomes [36–38]. By clustering cognitive trajectories, we aim to characterize different cognitive aging phenotypes and explore the interplay of brain biomarkers from EEG, MRI, and lifestyle risk factors within these phenotypes.

## Methods

This section outlines the data sources, biomarkers, processing steps, and approaches applied to cognitive trajectory clustering and statistical analysis. A summary of the methods used in the processing and analysis is provided in Fig. 1.

### Data

The present study is part of the University of Copenhagen Center for Healthy Aging (CEHA) neurobiology project, which aims to identify biomarkers related to differential trajectories of cognitive aging in otherwise healthy middle-aged men. This longitudinal cohort study spans almost five decades and includes all males born in the Copenhagen metropolitan area in 1953 [34]. The Metropolit cohort was incorporated into the Copenhagen Aging and Midlife Biobank (CAMB), which provides scores for both young adult draft board intelligence at around age 18 and late midlife intelligence at around age 57.

From the CAMB database, we recruited participants for three consecutive follow-up studies as part of the neurobiology project, conducted from 2010 to 2022. Subjects were included from all three cohorts, encompassing all baseline and follow-up assessments. Table 1 provides an overview of the participants in the three cohorts. In total, 582 participants were assessed at ages 57 to 68, with each participant having between one to three visits.

**Table 1:** Overview of participants in the three cohorts of the Metropolit data used in this study

Cohort	CEHA 1 (2010-2013)	CEHA 2 (2015–2020)	CEHA 3 (2020–2022)	Total
Participants (*N*)	211	503	113	582
		136 from CEHA 1	99 from CEHA 1–2	
			10 from CEHA 1	
Age (year)	57–59	61–67	66–68	57–68

### Covariates and biomarkers

To study cognitive changes over time, the study employs a variety of biomarkers obtained from different modalities. These include neuropsychological tests, MRI scans, EEG signals, and lifestyle risk factors. Neuropsychological assessments are used to evaluate cognitive functions such as memory, attention, processing speed, and executive function. These tests provide quantitative measures that can be tracked over time to identify changes in cognitive performance. Moreover, lifestyle and familial factors such as physical activity, alcohol consumption, and the presence of chronic conditions like type 2 diabetes, which are recorded through self-reported questionnaires and clinical assessments, are known to influence cognitive health and can interact with neurobiological markers to affect cognitive trajectories.

MRI scans offer detailed images of brain structure and function. Structural MRI can identify changes in brain volume and cortical thickness, which can help in understanding the neural correlates of cognitive decline and identifying early signs of neurodegeneration. Besides, EEG records electrical activity in the brain through sensors placed on the scalp, which can be analyzed for patterns that correlate with cognitive processes and potential abnormalities. EEG is particularly useful for assessing brain function in real time and can provide insights into neural connectivity and synchronization. A comprehensive list of the multimodal variables used in this study is presented in the significance plots found in the Appendix.

**Fig. 2:**
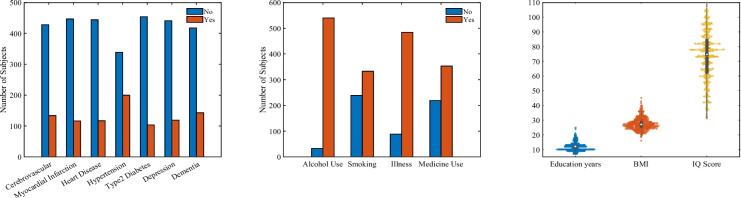
Grouped distribution of risk factors for the Metropolit study participants. The left panel presents a bar plot of familial health history, the middle panel displays a bar plot of lifestyle factors, and the right panel features a violin plot of individual measures

#### Neuropsychological tests

To assess cognitive functions, the neuropsychological test battery comprised a range of tests from different cognitive examinations including the CANTAB [7, 8]. These included motor screening (MOT), which assesses motor skills and provides a baseline for other tests; spatial recognition memory (SRM), measuring spatial memory; and pattern recognition memory (PRM), evaluating visual memory. The paired-associate learning (PAL) test is used to assess episodic memory and learning. The Stockings of Cambridge (SOC) test evaluates executive function and problem-solving abilities, while the reaction time (RTI) test measures the speed of response and processing. Lastly, the rapid visual processing (RVP) test assesses sustained attention and working memory.

In addition to the CANTAB tests, the study administered several other neuropsychological assessments. These included the Addenbrooke’s Cognitive Examination (ACE), which provides a broad assessment of cognitive functions; the Mini-Mental State Examination (MMSE), a widely used screening tool for cognitive impairment; the Symbol Digit Modalities Test (SDMT), which measures attention, visual scanning, and motor speed; and the Trail-Making Tests (TMT) A and B, which evaluate processing speed and task switching. Besides, retention and recall abilities were assessed using tests involving 15-word pairs.

#### Risk factors

Lifestyle and individual risk factors were assessed using a combination of categorical and real-valued measures. Binary variables included alcohol use, smoking, presence of illness, and medication use, each categorized as either “yes” or “no.” Real-valued individual measures included years of education, body mass index (BMI), and IQ scores obtained at age 12. Additionally, information about fathers’ occupations in 20 job categories and fathers’ educations in four categories were used, providing context for socioeconomic background. Familial health histories were also considered, with binary variables indicating the presence (“yes”) or absence (“no”) of conditions such as cerebrovascular disease, myocardial infarction, heart disease, hypertension, type 2 diabetes, depression, and dementia. Figure 2 shows the Metropolit participants’ binary and real-valued risk factors distribution.

#### MRI measures

MRI-based volumetry analysis was conducted using the FreeSurfer segmentation tool with the Desikan-Killiany atlas [39]. Regional brain volumes were calculated from T1-weighted scans acquired with a 3T Philips Achieva MRI scanner. The images had dimensions of 240 $\times$ 256 $\times$ 180 with an almost isotropic voxel size of 0.7 mm. The calculated volumes were corrected for intracranial volume (ICV) to adjust for individual differences in head size. A total of 35 regional brain volumes were analyzed for a detailed examination of structural brain changes, including the hippocampus, ventricles, white matter (WM), and cerebrospinal fluid (CSF).

In addition to the volumetry analysis, cortical thickness measurements were obtained using the FreeSurfer tool, which identifies the white matter to gray matter boundary and the pial surface with high accuracy [40]. This process involved segmenting the brain into distinct regions and measuring the thickness of the cerebral cortex per region, resulting in a comprehensive assessment across 34 cortical regions per hemisphere. This detailed examination included regions such as the fusiform gyrus, entorhinal cortex, supramarginal gyrus, and precuneus, among others.

#### EEG measures

EEG signals were recorded with a sampling frequency of 2 kHz using a 64-channel elastic Quick-Cap connected to a Compumedics Neuroscan bio-amplifier (SynAmps RT) with CURRY 7 Software. Electrodes were placed according to the international 10-20 system, with the online reference between Cz and CPz and the ground electrode between Fz and FPz. Participants were presented with visual stimulation at 8 Hz to assess steady-state visual evoked potential (SSVEP) power in the alpha band and at 36 Hz to assess SSVEP power in the gamma band.

Rubin’s vase, a complex high-contrast picture that requires cognitive processing but does not elicit an emotional response, was used as the stimulus target. Power spectrum analysis was performed using Welch’s method [41] to compute the peak power for each stimulation frequency. The spectral values were multiplied by their frequency to compensate for the 1/*f* shape of the EEG power spectrum. Finally, standardized values (*Z*-scores) of the power spectral densities (PSDs) were calculated for five main brain regions: temporal, frontal, parietal, central, and occipital. Further methodological details can be found in [42].

**Fig. 3:**
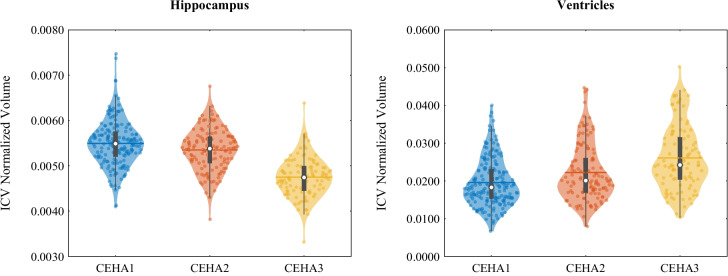
Violin plots of two regional MRI volumes at different visits of the participants

**Fig. 4:**
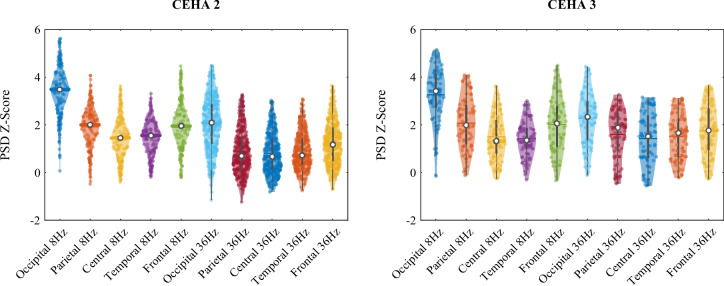
Violin plots of the regional EEG powers (logarithmic) at different visits of the participants

### Data processing

We first removed cognitive tests with fewer than 75 participants per visit and excluded those with constant scores within the [5, 95] percentile range across all visits. Next, we eliminated extreme outliers from the remaining scores using the interquartile range (IQR) method [43], filtering out values beyond five times the IQR. After this filtering process, 77 cognitive scores remained, including 70 from the CANTAB tests. Finally, we excluded cases with completely missing cognitive scores across all three visits, resulting in 526 participants for our analysis.

All 526 participants had valid data for the individual and lifestyle risk factors. However, some MRI and EEG data were missing due to participants’ absence, claustrophobia, or technical issues such as equipment malfunction. We applied similar filtering steps to remove missing measures, subjects, and extreme outliers from the MRI and EEG data and then matched these with the cognitive scores. This process yielded 35 normalized anatomical brain volumes from 414 T1-weighted scans of 190 participants, each with up to three visits. For the EEG data, we obtained 10 normalized regional measures from 64 channels of 349 participants, each with up to two visits. Figures 3 and 4 illustrate the violin plots of the regional MRI volumes and EEG powers used in this study.

#### Cognitive summary

To effectively capture major cognitive changes over time, we combined and reduced the dimensionality of all neuropsychological test scores. We used autoencoders comprising two fully connected layers [44], which are artificial neural networks designed for unsupervised learning. Autoencoders provide an efficient alternative to principal components analysis by using learnable parameters and nonlinear functions. Autoencoders encode high-dimensional input data into abstract latent variables while filtering out insignificant information. The network then reconstructs the input data from these latent variables, minimizing reconstruction error through iterative training. This process allows the autoencoder to extract meaningful features that summarize the cognitive data.

To ensure optimal performance, we applied tenfold cross-validation to tune the network’s hyperparameters and determine the appropriate size of the latent space. The resulting latent features served as cognitive summaries for the participants. Our validation results showed that using 65% of the original input size was sufficient for optimal reconstruction of the cognitive data, resulting in 50 latent features. This method provided a comprehensive and efficient way to summarize cognitive changes, facilitating trajectory clustering and further analysis of the relationship between cognitive performance and other biomarkers.

#### Trajectory clustering

While several individual measures are associated with cognitive changes, no single cognitive test is sufficiently accurate in determining aging trajectories at the individual level. It is necessary to move from separate, univariate prediction models to combined multivariate analyses to reduce variability and increase accuracy. To achieve this, we proposed clustering longitudinal cognitive data into homogeneous groups of participants who share similar trajectories using a temporal k-means clustering algorithm [45] with temporal centroids and multivariate dynamic time warping (DTW) distance. This approach measures the distance between two multivariate temporal data points, allowing for comparing temporal signals with varying lengths or missing values.

We applied the temporal k-means clustering algorithm to the summarized multivariate cognitive scores, resulting in four distinct groups representing different patterns of cognitive performance changes over time. To handle the temporal alignment of sequences of varying lengths, we employed the DTW distance metric to calculate the optimal alignment path by comparing all possible pairs across two sequences, aligning similar patterns regardless of time-point differences. Next, *k*-means clustering was applied to obtain *k*-nearest neighbor trajectories and group the resulting centroids. This method enabled the comparison of trajectories without imputation by focusing on overall shape and trend rather than exact temporal correspondence.

The optimal number of groups was selected by minimizing the Davies-Bouldin index, which was used as the criterion for evaluating the quality of the trajectory clustering. Our hypothesis posited that there would be four distinct types of cognitive trajectories among the participants. These trajectories would include high-performing and low-performing groups, as well as those exhibiting either stable or declining cognitive function over time. This method enabled us to better understand and characterize the different trajectories of cognitive aging within our cohort, providing a more comprehensive view of cognitive changes over time and helping us to detect at-risk or early symptomatic patients.

### Statistical analysis

To analyze significant differences between groups, we employed various statistical tests depending on the data type. For continuous variables, we used the nonparametric Wilcoxon rank-sum test to compare two groups (e.g., high-performing vs. low-performing), and the Kruskal-Wallis test for comparisons involving multiple groups. For categorical variables, Fisher’s exact test was used when dealing with binary outcomes, while the chi-square test was applied to study the ratios of categorical variables across multiple groups. A significance threshold of $p < 0.05$ was used to determine the outcomes of the hypothesis tests.

## Results

### Cognitive groups

**Fig. 5:**
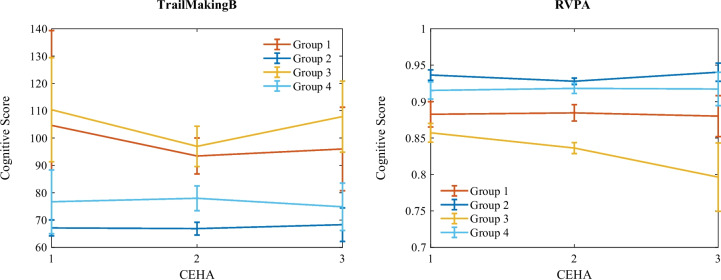
Two cognitive test trajectories grouped using temporal clustering. The error bars represent the 95% confidence interval for the population standard deviation per visit. The RVPA scores reflect the subject’s sensitivity in identifying target stimuli, while the Trail Making B test is measured by the time (in seconds) taken to complete the task. Hence, Group 1 and Group 3 represent low-performing patients, while Group 2 and Group 4 correspond to high-performing individuals

The clustering analysis identified four distinct cognitive groups among the participants: Group 1 with 69 participants, Group 2 with 256 participants, Group 3 with 65 participants, and Group 4 with 136 participants. Figure 5 illustrates the cognitive trajectories of these groups, represented by two different test scores. As can be seen, Group 1 and Group 3 represent low-performing patients, with Group 1 displaying stable trajectories and Group 3 showing a decline. Conversely, Group 2 and Group 4 correspond to high-performing individuals, with Group 2 exhibiting higher cognitive levels and Group 4 showing lower cognitive levels. Note that these group labels were determined based on a combination of all cognitive scores.

The obtained trajectory groups highlight different aspects of cognitive performance. Groups 1 and 3 refer to low-performing individuals, while Groups 2 and 4 represent more normal cognitive functioning. Specifically, the trajectories of the MMSE and ACE scores aligned, with the high achiever groups (Groups 2 and 4) showing higher MMSE levels. The high-performing groups had significantly lower and stable trail-making times and higher and stable SDMT scores over time. Conversely, the lower functioning groups (Groups 1 and 3) had significantly higher trail-making scores and lower SDMT scores and showed a decline over time in both tests. Interestingly, one high achiever group (Group 2) displayed an almost stable trajectory and significantly lower levels in the 15-word pairs and Retention tests compared to the other groups that had higher scores initially and decreased over time.

The PAL test results indicated that Group 1 had significantly lower memory scores, more errors and trials, and fewer completed stages. This low-performing group also performed worse in the PRM and SRM tests. In contrast, Group 3 began with higher levels and declined over time. The errors in the RTI test were more stable and lower in the high-performing groups compared to the low-performing ones. In the RVP test, the high-performing groups maintained stable scores significantly different from the low-performing groups. The low-performing groups had significantly lower levels of RVPA, hits, and correct rejections, with Group 3 showing a rapid decline. They also exhibited more misses, false alarms, and latency. In the SOC test, the low-performing groups solved fewer problems, made more moves, and took longer initial thinking time, with these metrics declining over time compared to the high-performing ones.

**Fig. 6:**
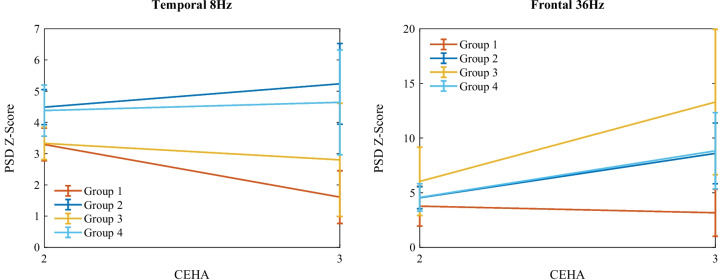
Two EEG measure trajectories (logarithmic) grouped using the cognitive clusters. The error bars represent the 95% confidence interval for the population standard deviation per visit

**Fig. 7:**
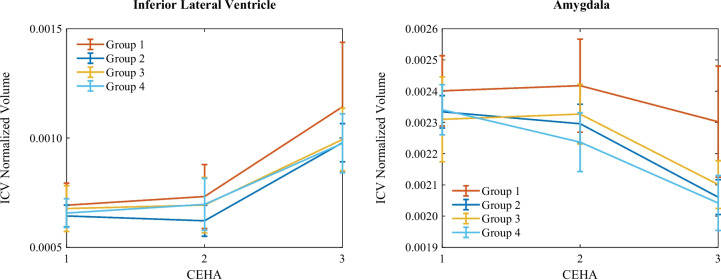
Two MRI volume trajectories grouped using the cognitive clusters. The error bars represent the 95% confidence interval for the population standard deviation per visit

The statistical analysis results reveal statistically significant differences in most cognitive tests between the various clustered groups. The analysis highlights the robustness of our clustering approach in differentiating between cognitive performance trajectories, providing strong evidence that the identified groups are distinct in terms of their cognitive abilities. This significant differentiation underscores the importance of using comprehensive, multivariate methods for accurately capturing the complexity of cognitive aging and identifying meaningful patterns within the data.

### Grouped EEG measures

EEG-evoked power parameters were analyzed according to the cognitive clusters. Two sample measures of EEG powers grouped using the obtained cluster labels, are illustrated in Fig. 6. The grouping revealed that the low-performing Group 1 had the lowest evoked power in almost all brain regions for both EEG alpha and gamma frequency bands. Their trajectories showed severe declines in the alpha frequency band over time. Conversely, the low-performing Group 3 exhibited the greatest rates of increase over time in almost all brain regions of the gamma frequency band.

The group differences in EEG-evoked power levels and changes were more pronounced in the parietal and temporal regions for both alpha and gamma frequencies. These findings highlight differences in neuronal synchronization across cognitive clusters and suggest varying trajectories of neuronal synchronization with aging. As SSVEP reflects how efficiently neurons shift from spontaneous to stimulus-driven rhythmic activity, these results indicate that SSVEP-elicited activation patterns may help differentiate cognitive trajectories by measuring neuronal synchronization ability.

The statistical tests were performed using the Kruskal-Wallis test for multiple groups. If the two low-performing and two high-performing groups were merged into two broader categories, we could identify even more regions with significant differences. This suggests that while our current clustering approach provides detailed insights into specific group differences, a more generalized grouping could highlight broader patterns of EEG-evoked power changes associated with cognitive impairment and normal cognitive aging.

**Table 2:** Statistics of familial and socioeconomic risk factors for the Metropolit participants, grouped by cognitive clusters

	Hypertension	Diabetes	Depression	Dementia	Father’s education
	(yes/no)	(yes/no)	(yes/no)	(yes/no)	(primary/middle/high)
Group 1	27/39	10/59	11/58	19/50	17/20/29
Group 2	88/153	51/201	63/187	65/184	41/90/102
Group 3	26/36	17/44	16/48	15/49	14/17/27
Group 4	44/84	16/116	20/112	38/96	29/45/51

**Table 3:** Statistics of individual and lifestyle risk factors for the Metropolit participants, grouped by cognitive clusters

	Alcohol use	Smoking	Illness	School years	IQ score
	(yes/no)	(yes/no)	(yes/no)	mean±SD	mean±SD
Group 1	65/4	40/29	58/11	10.76±2.59	65.09±15.60
Group 2	243/13	147/109	213/43	12.27±2.84	79.85±14.27
Group 3	63/2	41/24	58/7	11.05±2.56	65.27±16.65
Group 4	128/8	78/58	114/22	11.52±2.66	71.91±14.43

### Grouped MRI measures

MRI measurements were also analyzed according to the cognitive clusters. Two samples of MRI volumes grouped using the obtained cluster labels are illustrated in Fig. 7. The analysis revealed that, in contrast to the high-performing groups, the low-performing Group 1 exhibited relatively lower rates of change over time. In contrast to typical aging, this group also showed higher volumes in several brain regions, including the ventricles, caudate, putamen, amygdala, brain stem, corpus callosum, total brain, cortex, cerebral white matter, gray matter, and supratentorial regions.

There was a general reduction in brain volume across most regions of all groups such as the amygdala and brain stem, accompanied by notably increased volumes of ventricles and CSF. We observed that neocortical and basal ganglia structures such as the caudate and putamen underwent more pronounced atrophy earlier in the observation period while limbic and subcortical regions like the hippocampus and amygdala showed more rapid changes at later stages. Additionally, in some regions such as the lateral ventricle, third/fourth ventricle, accumbens area, optic chiasm, corpus callosum, and total brain, the rate of change remained almost constant over time.

The low-performing groups showed a higher thickness in several regions, including the insula, temporal pole, frontal pole, supramarginal gyrus, superior temporal gyrus, superior parietal lobule, superior frontal gyrus, precuneus, precentral gyrus, paracentral lobule, middle temporal gyrus, medial orbitofrontal cortex, lingual gyrus, lateral orbitofrontal cortex, lateral occipital cortex, inferior temporal gyrus, inferior parietal lobule, fusiform gyrus, entorhinal cortex, cuneus, and caudal middle frontal gyrus. This unexpected result may reflect decoupled processes of brain volume reduction and cortical thinning, suggesting potential compensatory mechanisms or other factors contributing to these observations.

There was a general trend of decreasing cortical thickness over time across most regions, but the rate of change varied across different regions and periods. In some regions like the insula, superior temporal gyrus, and middle temporal gyrus, the rate of thickness change remained constant. In regions like the precentral gyrus and supramarginal gyrus, changes were more pronounced in the earlier visits, while in regions like the entorhinal cortex, fusiform gyrus, and temporal pole, the rate of change accelerated in the later stages. These regional differences suggest complex and heterogeneous processes of cortical thinning and brain atrophy during aging, influenced by cognitive performance levels and other factors such as vascular health or neurodegenerative changes.

**Fig. 8:**
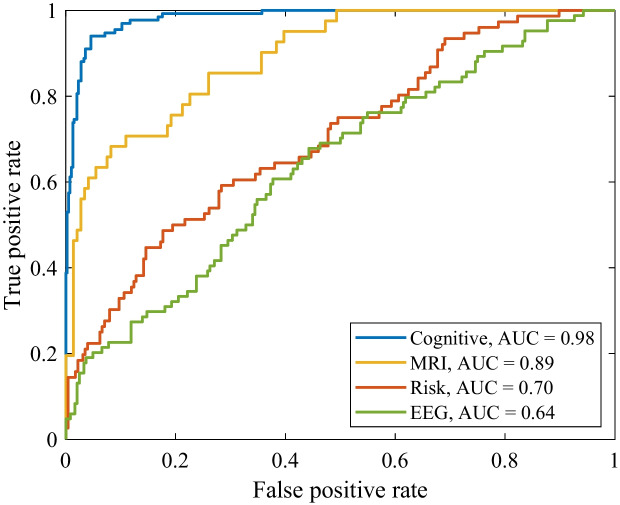
ROC for various modality markers from the Metropolit participants used in detecting patients with low-performing cognitive trajectories from high-performing ones

### Grouped risk factors

Familial, individual, and socioeconomic risk factors were also analyzed based on the cognitive clusters identified in the study. Tables 2 and 3 provide detailed statistics of both familial and individual risk factors within each cognitive cluster. Our analysis revealed distinct patterns of risk factors associated with each cognitive group. The two high-performing groups (Groups 2 and 4) exhibited higher IQ scores, particularly Group 2, and longer years of education than low-performing groups. These groups also demonstrated lower percentages of familial hypertension, smoking, and illness. Specifically, Group 4 had the lowest incidence of familial type 2 diabetes and alcohol use, while Group 3, one of the low-performing groups, showed the highest percentages of these factors among others.

Additionally, the employment status and educational background of the participants’ fathers provided further insights. The fathers of participants in the high-performing groups were more likely to be employed as civil servants or hold superior job positions like directors, with Group 2 showing a particularly high percentage of fathers in such roles. This correlation suggests that higher cognitive scores are associated with higher IQ levels, superior paternal occupation, and lower incidences of smoking, alcohol use, and comorbidities. These findings underscore the interplay between cognitive performance and various risk factors. Participants in the high-performing cognitive groups generally had a more favorable risk profile, which included higher educational attainment, healthier lifestyle choices, and less familial predisposition to certain health conditions. Conversely, the low-performing cognitive groups were characterized by a higher prevalence of adverse risk factors, such as lower IQ scores, lower education, higher rates of smoking and alcohol use, and greater familial history of hypertension and diabetes.

**Fig. 9:**
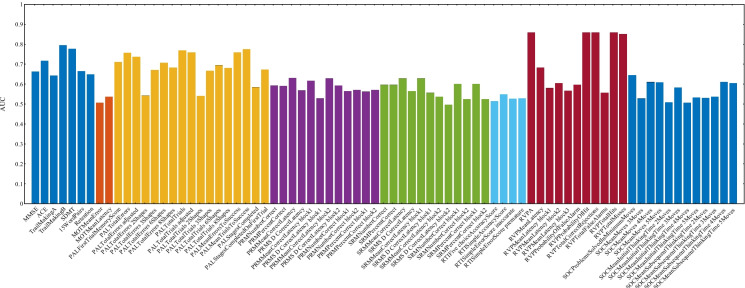
AUC for each cognitive score in detecting patients with low-performing cognitive trajectories from high-performing ones

### Cognitive decline prediction

Finally, we aimed to show the utility of cognitive labels obtained through unsupervised clustering by predicting cognitive decline. To achieve this, we combined Groups 2 and 4 into a high-performing group and Groups 1 and 3 into a low-performing group, creating a binary classification task. Next, we applied logistic regression to distinguish between these two groups with a multivariate analysis incorporating multiple biomarkers from each domain independently, including cognitive tests, MRI measures, EEG powers, and risk factors. We used the first available data points per subject per modality as multivariate predictors. Cognitive scores were primarily used to validate the labels generated through unsupervised clustering by performing supervised classification on the cognitive groups. Figure 8 presents the receiver operating characteristic (ROC) curves for the different modality markers in predicting cognitive decline.

As anticipated, cognitive test scores yielded the highest classification accuracy in distinguishing between the cognitive groups with an area under the curve (AUC) of 0.98. This highlights the effectiveness of cognitive tests in differentiating between high-performing and low-performing individuals based on their cognitive trajectories. Besides, MRI measures demonstrated a strong predictive capability with an AUC of 0.89. This finding underscores the importance of structural brain changes as significant indicators of cognitive impairment over time. Moreover, these results affirm the potential of using unsupervised clustering methods to label patients based on their cognitive trajectories. By integrating multimodal biomarkers, the model can leverage comprehensive data to accurately classify individuals, providing a robust framework for early identification and monitoring of cognitive impairment.

To evaluate the predictive power of each cognitive score for identifying cognitive decline, we applied logistic regression using the combined group labels and performed a univariate analysis. Figure 9 depicts the AUCs for the various cognitive tests in predicting cognitive decline. Notably, the rapid visual processing tests achieved the highest AUCs, indicating superior predictive performance, followed by the paired-associate learning tests and general neuropsychological assessments such as the trail-making times and SDMT.

## Discussion

In this study, we employed deep machine learning algorithms to explore the impact of individual, familial, and socioeconomic risk factors on cognitive decline within the Metropolit cohort. Our approach involved clustering multivariate cognitive trajectories into four homogeneous groups based on their cognitive performance and examining the associated risk factors and structural and functional changes. Our primary findings revealed that type 2 diabetes mellitus, lower education, alcohol consumption, IQ, and father’s occupation are significant combined risk factors for cognitive decline, particularly evident in Group 3. These results align with existing literature, where educational attainment has been consistently linked to higher cognitive levels and a slower rate of cognitive decline across varying academic backgrounds and income levels [46].

The four cognitive groups were derived using the k-means clustering method with dynamic time warping distance, which captures temporal behavior similarities across all trajectories rather than individual score levels. This approach allowed us to observe distinct cognitive changes across various tests, such as the differences between the low-performing stable and low-performing declining groups in the Trail Making and RVPA tests. Clustering was performed on a comprehensive set of 77 test scores, integrating all these markers to form the groups. For clarity, we illustrated the groups using only two cognitive test scores, highlighting their average trajectories and deviations. It is important to note that the clustering was based solely on cognitive test scores, not MRI and EEG measures.

The groups were defined based on three visits from 2010 to 2022. Hence, the variations might reflect individual differences in cognitive aging or indicate cognitive development earlier in life before 2010. There are significant individual differences in cognitive performance in young adulthood, and these differences tend to remain stable across a large part of the lifespan [47]. Consequently, it makes sense that we found young IQ to differ among the four groups, and that education levels also varied as education is known to be strongly associated with cognitive ability and may influence cognitive development [48]. However, the evidence on the effects of education on cognitive decline is inconsistent [49]. There is extensive literature on the relationship between early-life intelligence and health-related behaviors, morbidity, and mortality [50], as well as the association between offspring intelligence and parental occupation and social status [51]. Our findings align with this literature, underscoring the importance of considering early-life cognitive abilities and socioeconomic factors when studying cognitive aging trajectories.

Education is a well-established factor in cognitive reserve, providing a buffer against cognitive decline by promoting neural plasticity and cognitive strategies that mitigate the impact of aging and neuropathological changes [52]. Consistent with this literature, our study found that higher education is correlated with better cognitive function across various domains. However, our findings also indicated that other risk factors, such as diabetes mellitus and higher alcohol consumption, contribute to a more rapid decline in cognitive performance. This multifaceted impact highlights the importance of considering a combination of educational, lifestyle, and health-related factors when assessing cognitive aging.

Diabetes mellitus has been consistently linked to cognitive impairment, likely due to the disease’s effects on cerebrovascular health and glucose metabolism in the brain [53]. Similarly, high alcohol consumption has been associated with neurodegeneration and cognitive deficits, emphasizing the need for moderation to maintain cognitive health [54]. Furthermore, the role of socioeconomic status, encapsulated by the father’s occupation in our study, supports the theory that early-life socioeconomic conditions influence cognitive health later in life, as they are often correlated with access to resources, healthcare, and overall life stability [55].

Our clustering method identified a subset of subjects in Group 1 who exhibited lower education levels and cognitive performance at ages 57-59 but had no comorbidities such as diabetes mellitus or high alcohol intake. These individuals demonstrated a more stable cognitive performance over time, highlighting the complexity of cognitive decline processes. This finding aligns with recent studies that have reported mixed results regarding the association between low to moderate alcohol consumption and cognitive functions. Some studies suggest that low to moderate alcohol intake is associated with better global cognition scores, even after clustering participants into distinct cognitive function trajectories [56].

Our approach, which clustered participants into four distinct groups based on their longitudinal cognitive performance, revealed significant variations in cognitive outcomes among the low-achiever groups. This underscores the multifactorial nature of cognitive decline, suggesting that factors beyond education and comorbidities, such as genetic predispositions, lifestyle choices, and environmental influences, play crucial roles in determining cognitive trajectories. This nuanced understanding emphasizes the importance of personalized interventions and the need for further research to unravel the complex interplay of factors contributing to cognitive aging.

The longitudinal observation of brain structure indicated that volumes of neocortical and basal ganglia structures such as the caudate and putamen decline earlier than those of the limbic and subcortical structures like the amygdala and hippocampus. It is known that cognition and emotion are distinct processes facilitated by different brain regions, with the amygdala playing a key role in emotion and the prefrontal cortex being crucial for cognitive functions. The functional interactions between the amygdala and prefrontal cortex mediate the emotional influences on cognitive processes, such as decision-making, and the cognitive regulation of emotion [57]. The early decline in neocortical volumes, in contrast to the relatively stable subcortical volumes, might explain why cognitive symptoms often precede emotional disturbances in neurodegenerative diseases.

Brain aging and dementia are associated with various morphological changes, including cortical thinning, loss of white and gray matter volume, ventricular enlargement, and decreased gyrification, all contributing to cognitive changes [58]. The cortical decline alongside the relative preservation of paleocortical structures likely contributes to the behavioral and emotional symptoms observed in dementia. Although none of our participants exhibited clinical symptoms of dementia, these structural changes were detectable through MRI analysis and could help us differentiate between the neurological conditions. Regions such as the insula, temporal pole, and superior temporal cortex displayed more pronounced thinning over time, whereas other regions, like the lateral occipital and peri-calcarine cortices, showed increases in thickness. This differential pattern of cortical thinning and growth highlights the complex nature of brain conditions.

The finding that low-performing groups demonstrated higher cortical thickness was unexpected, as cognitive decline is generally associated with cortical thinning, particularly in aging and neurodegenerative conditions [59]. One potential explanation is that this increased cortical thickness could reflect compensatory mechanisms [60]. In some cases, the brain may attempt to compensate for cognitive deficits by increasing the size of certain regions involved in cognitive control or decision-making. This aligns with findings in neuroplasticity, where cortical hypertrophy can occur in response to functional inefficiencies in the early stages of cognitive decline [61]. This compensatory mechanism has been observed in other neurodegenerative studies as well, where cortical thickening in regions like the frontal and temporal lobes has been linked to compensatory plasticity early in cognitive decline, which may later give way to atrophy as the disease progresses [62, 63]. Another possibility relates to developmental or early-life factors, where individuals with lower educational attainment or early-life cognitive performance may retain increased cortical thickness later in life [64]. Variations in lifestyle factors and genetic influences may also contribute to these structural differences, as these factors are known to impact cortical thickness and cognitive outcomes [65].

The EEG-evoked power responses are critical indicators of the brain’s ability to synchronize neural networks. In our study, changes in SSVEP power were most pronounced in the parietal and temporal regions, with the occipital region remaining relatively stable. Notably, our low-performing Group 1 exhibited a significant decline in alpha power over time in the temporoparietal region, whereas low-performing Group 3 did not show a corresponding electrographic decline despite a greater cognitive performance decline. Interestingly, Group 3 even showed an increase in frontotemporal SSVEP power at gamma frequency, despite their cognitive decline.

The discrepancy between cognitive performance and alpha power changes between Groups 1 and 3 may be due to underlying neurobiological differences in how aging affects neural networks. Alpha oscillations tend to decline with aging due to neural network inefficiency, particularly in areas involved in attention and working memory [66]. Group 1’s decrease in alpha power could reflect this expected decline, whereas Group 3’s stability in alpha power despite various cognitive reductions, could suggest that they have inherently lower baseline neural efficiency where further declines are less evident. Additionally, cognitive reserve modulated by factors like education and lifelong mental engagement may play a role in stabilizing neural oscillations even as cognitive performance changes, offering compensatory mechanisms that preserve alpha power in certain aging populations [67].

On the contrary, the high-performing Groups 2 and 4 demonstrated slight increases in evoked frontotemporal power at both alpha and gamma stimulations. This observation supports the posterior-anterior shift in aging (PASA), a consistent finding from functional neuroimaging studies of cognitive aging. This shift, characterized by decreased occipital activity coupled with increased frontal activity, is typically attributed to functional compensation mechanisms [68]. Our findings align with our previous study, where we observed relatively stable cognitive performance but a notable posterior-anterior shift of evoked gamma power between the ages of 63–68 years [42]. During this period, progressive atrophy of the hippocampus was observed and correlated with visual and verbal memory decline [69].

The reduced occipital activity coupled with increased frontal activity underscores the brain’s compensatory mechanisms in response to aging. This PASA effect has been corroborated by numerous MRI and single-photon emission computed tomography (SPECT) studies, which have indicated the preservation of the occipital cortex in Alzheimer’s dementia [17]. EEG-based studies have also supported these findings, suggesting that while occipital activity diminishes with age, frontal regions become more engaged to maintain cognitive functions [70].

## Conclusion

In this present study, we tested how machine learning algorithms can help to find preclinical predictors of cognitive decline and reserve in different homogeneous groups of subjects in our large cohort. By clustering our data into four groups of participants, we could observe that cognitive changes during advanced age, structural changes on MRI, and functional changes on SSVEPs had different patterns in different groups. We could detect predictive patterns regarding lifestyle, illnesses, education, and outcomes on MRI and SSVEPs with the clustered groups. This was an effective way to capture different risk factors and relevant diagnostic tests to detect cognitive decline and label big cohorts to find at-risk patients at a very low cost and long before clinical signs.

In this study, we utilized machine learning algorithms to identify preclinical predictors of cognitive decline and cognitive reserve within a heterogeneous cohort devoid of predefined labels. By clustering the participants into four distinct groups, we were able to observe unique patterns of cognitive, structural, and functional changes associated with aging. Our findings revealed predictive patterns related to lifestyle, familial, and socioeconomic risk factors as observed through MRI and EEG analyses. This methodology enhanced our understanding and characterization of the diverse trajectories of cognitive aging, providing a comprehensive view of cognitive changes over time and aiding in the identification of at-risk or early symptomatic individuals.

The robustness of our clustering approach was demonstrated through its ability to differentiate cognitive performance effectively. The significant differentiation between the identified groups underscores the necessity of comprehensive, multivariate methods to accurately capture the complexity of cognitive aging and to identify meaningful patterns within the data. This approach provided strong evidence that the groups identified are distinct in terms of their cognitive abilities, reinforcing the value of using advanced clustering techniques in cognitive research.

Our analysis also highlighted specific brain regions where EEG-evoked power levels and changes were most pronounced, particularly in the parietal and temporal regions for both alpha and gamma frequencies. This offers information about neuronal synchronization ability, which is indirectly relevant to cognitive performance. Hence, assessing activation patterns from the SSVEP may help to differentiate between different cognitive trajectories. Furthermore, MRI measures revealed significant differences in the levels and changes across various brain regions, with the low-performing groups exhibiting lower rates of change over time and higher volumes in several areas compared to the high-performing groups. Notably, the low-performing groups showed increased cortical thickness in specific regions, further highlighting the complexity of the cognitive decline process.

Participants in the high-performing cognitive groups generally presented with a more favorable risk profile, including higher educational attainment, healthier lifestyle choices, superior fathers’ jobs, and less familial predisposition to certain health conditions. In contrast, the low-performing cognitive groups were characterized by a higher prevalence of adverse risk factors such as lower IQ scores, shorter educational periods, higher rates of smoking and alcohol use, and a greater familial history of hypertension and type 2 diabetes. Additionally, our study found that MRI measures demonstrated a strong predictive capability with an AUC of 0.89, emphasizing the importance of structural brain changes as significant indicators of cognitive impairment over time.

## Figures and Tables

**Fig. 10:**
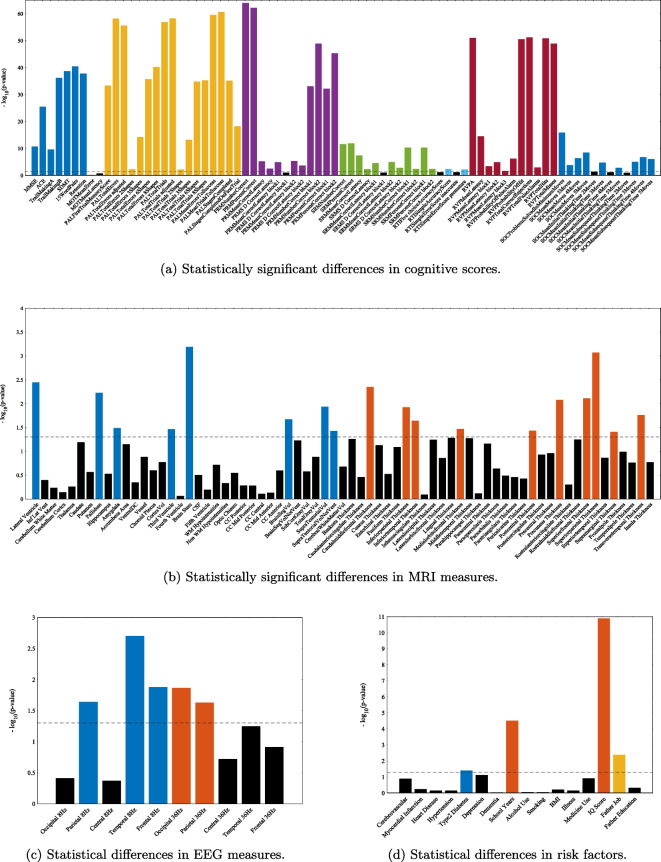
Statistically significant differences in different modality markers between the clustered groups of the Metropolit cohort with logarithmic *p*-values per biomarker. The dashed line indicates the 0.05 threshold, while the black bars represent non-significant markers

## Data Availability

Data is not publicly available due to ethical restrictions and participant privacy concerns. Requests for access to the data may be submitted to Prof. Martin Lauritzen for review and approval.
